# Optical fibers for endoscopic high-power Er:YAG laserosteotomy

**DOI:** 10.1117/1.JBO.26.9.095002

**Published:** 2021-09-13

**Authors:** Lina M. Beltrán Bernal, Ferda Canbaz, Salim E. Darwiche, Katja M. R. Nuss, Niklaus F. Friederich, Philippe C. Cattin, Azhar Zam

**Affiliations:** a University of Basel, Department of Biomedical Engineering, Faculty of Medicine, Biomedical Laser and Optics Group, Allschwil, Switzerland; b University of Zürich, Musculoskeletal Research Unit, Zürich, Switzerland; c University of Zürich, Center for Applied Biotechnology and Molecular Medicine, Zürich, Switzerland; d University of Basel, Department of Biomedical Engineering, Faculty of Medicine, Center of Biomechanics and Biocalorimetry, Allschwil, Switzerland; e University of Basel, Department of Biomedical Engineering, Faculty of Medicine, Center for medical Image Analysis and Navigation, Allschwil, Switzerland

**Keywords:** laser ablation of bone, Er:YAG laser, optical fiber, germanium oxide fiber, sapphire fiber, zirconium fluoride fiber, hollow-core silica waveguide, deep bone ablation, minimally invasive laserosteotomy

## Abstract

**Significance:** The highest absorption peaks of the main components of bone are in the mid-infrared region, making Er:YAG and ${\mathrm{CO}}_{2}$ lasers the most efficient lasers for cutting bone. Yet, studies of deep bone ablation in minimally invasive settings are very limited, as finding suitable materials for coupling high-power laser light with low attenuation beyond 2  μm is not trivial.

**Aim:** The first aim of this study was to compare the performance of different optical fibers in terms of transmitting Er:YAG laser light with a 2.94-μm wavelength at high pulse energy close to 1 J. The second aim was to achieve deep bone ablation using the best-performing fiber, as determined by our experiments.

**Approach:** In our study, various optical fibers with low attenuation (λ=2.94  μm) were used to couple the Er:YAG laser. The fibers were made of germanium oxide, sapphire, zirconium fluoride, and hollow-core silica, respectively. We compared the fibers in terms of transmission efficiency, resistance to high Er:YAG laser energy, and bending flexibility. The best-performing fiber was used to achieve deep bone ablation in a minimally invasive setting. To do this, we adapted the optimal settings for free-space deep bone ablation with an Er:YAG laser found in a previous study.

**Results:** Three of the fibers endured energy per pulse as high as 820 mJ at a repetition rate of 10 Hz. The best-performing fiber, made of germanium oxide, provided higher transmission efficiency and greater bending flexibility than the other fibers. With an output energy of 370 mJ per pulse at 10 Hz repetition rate, we reached a cutting depth of 6.82±0.99  mm in sheep bone. Histology image analysis was performed on the bone tissue adjacent to the laser ablation crater; the images did not show any structural damage.

**Conclusions:** The findings suggest that our prototype could be used in future generations of endoscopic devices for minimally invasive laserosteotomy.

## 1 Introduction

In the field of osteotomy, the use of lasers has been studied for several years;^1^^–^^8^ however, some early clinical studies showed severe collateral damage and a prolonged healing process.^9^ In response, efforts have been made to optimize laser systems intended for laserosteotomy. For example, modern Er:YAG laser systems have been used to remove intraoral hard tissue in humans, without showing damage in subsequent histological analyses.^10^ More recent studies have shown how the use of new irrigation and temperature feedback detection systems for Er:YAG laserosteotomy can help achieve safe, deep bone ablation.^11^ Other results from a robotic free-space laser device, CARLO^®^, based on an Er:YAG laser, show potential for use in osteotomy applications. The CARLO laser device was used for the first clinical bone surgery worldwide, where the functionality of the patient’s jaw was improved. After years of investigation in the field, the device was used in July 2019 for an *in vivo* mid-face osteotomy at the Department of Oral Maxillofacial Surgery, University Hospital Basel, Switzerland.^7^^,^^12^^,^^13^

Several aspects make lasers attractive for tissue ablation. It is a safer procedure with respect to preventing bacterial and viral infection during surgery because of the contactless nature of the laser–tissue interaction. Lasers also make it easier to achieve the flexible cuts (various shapes and curves) desired during surgery, especially for cutting hard tissue. Although surgery with conventional mechanical tools has evolved to be as minimally invasive as possible, the flexibility provided by these tools remains limited.^14^ Endoscopic devices have emerged for use in minimally invasive procedures. These devices provide solutions for diagnosis and microsurgeries inside the body where other tools cannot reach.^15^^,^^16^ For endoscopic laser surgery involving hard tissues, some studies have shown the benefits of using fiber-coupled or fiber-based lasers.^17^^,^^18^ Lasers working from 600 nm up to 2  μm can be easily coupled in low-OH silica fibers due to their low attenuation in this wavelength region. For instance, a Ho:YAG laser at a wavelength of 2.12  μm has been widely used for lithotripsy;^19^ it can be used in endoscopic applications by means of silica fibers, as can the thulium laser at a wavelength of 1.908  μm.^20^ For endoscopic surgical applications requiring deeper hard-tissue ablation, such as a typical maxillofacial surgery on the jaw or during knee surgery, a laser delivering more energy per pulse through the optical fiber is needed. We return to the Er:YAG laser, which has shown outstanding results for deep bone ablation.^10^^,^^11^ Throughout the history of fiber research, finding materials for coupling laser light with low attenuation around 3  μm has not been trivial. Today, however, some materials, such as sapphire, fluoride,^18^ and germanium oxide fibers,^17^ show encouraging results regarding the transmission of infrared (IR) light close to 3  μm. The main purpose of this study was to investigate some of the fibers known to efficiently transmit Er:YAG laser energy at a wavelength of 2.94  μm. For this study, we chose four different fibers to couple an Er:YAG laser. Transmission efficiency, resistance to high-power laser energy, and bending flexibility are the key criteria to consider when selecting a fiber for endoscopic laserosteotomy, especially for deep bone ablation.

## 2 Materials and Methods

### 2.1 Laser Source and Optical Fibers

The laser source used in this study was a microsecond high-power Er:YAG laser (Syneron Litetouch) with wavelength λ=2.94  μm. The energy per pulse of the laser could be set within the range 100 to 820 mJ, with a repetition rate range of 1 to 50 Hz and a pulse duration range of 100 to 400  μs. We studied the performance of four different fibers in terms of (1) the transmission efficiency and its stability over time; (2) the input tip temperature; and (3) the bending stability of the fibers over time. The fibers chosen for our study are known to have high transmission in the mid-IR wavelength range. To compare performance, we used fibers with similar core sizes and a 1-m length. The glass-based fibers were made of germanium oxide (${\mathrm{GeO}}_{2}$), sapphire (${\mathrm{Al}}_{2}O_{3}$), zirconium fluoride glass (${\mathrm{ZrF}}_{4}$), and a hollow-core silica waveguide, respectively. Table 1 displays the main properties of the fibers used.

**Table 1:** Properties of the optical fibers used.

	Germanium (${\mathrm{GeO}}_{2}$)	Sapphire (${\mathrm{Al}}_{2}O_{3}$)	Fluoride (${\mathrm{ZrF}}_{4}$)	Hollow-core silica
Core size (μm)	450	425	400	500
NA	0.25	0.12	0.2	0.025
Attenuation (dB/m)	0.5	0.25	0.015	1.5
Min. bending rad (cm)	4	8	4.5	15
Manufacturer	Infrared Fiber Systems	Laser Components	Le Verre Fluoré	Laser Components

**Fig. 1:**
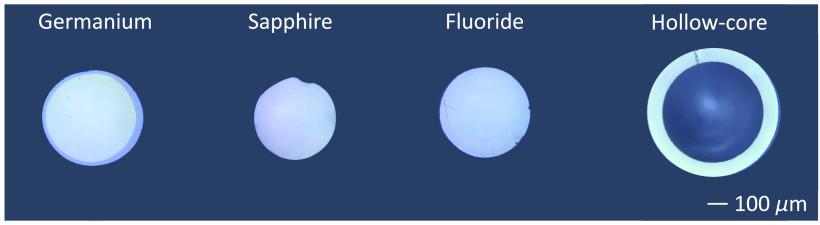
Microscopic images of the fibers’ polished surfaces.

“Germanium” fibers consist of a germanium oxide (${\mathrm{GeO}}_{2}$) glass core, a glass cladding, a polyamide coating, and a thermoplastic coating in the external layer. “Sapphire” (${\mathrm{Al}}_{2}O_{3}$) fibers have neither cladding nor sheathing. “Fluoride” fibers are made of a zirconium fluoride (${\mathrm{ZrF}}_{4}$) glass core and have a double cladding. The second cladding is made of low index resin. It has an external polyacrylate sheathing and germanate end caps. End-capping is a technology in which a different material is spliced into the fiber tip to reduce the power density at the end tips, thereby decreasing the heat as well. The inner wall of “hollow-core” fiber is made of a silver iodide (AgI) reflector, covered by a silica tube and an external acrylate buffer. All of the fibers are commercially available, except for the fluoride one. Currently, fluoride fibers are in a testing phase. Figure 1 shows the end tips of the fibers when they are polished. The end tips were observed with a fiber inspection scope (Thorlabs, FS200) and images were taken using a mobile phone camera.

### 2.2 Coupling the Laser into the Fibers

**Fig. 2:**
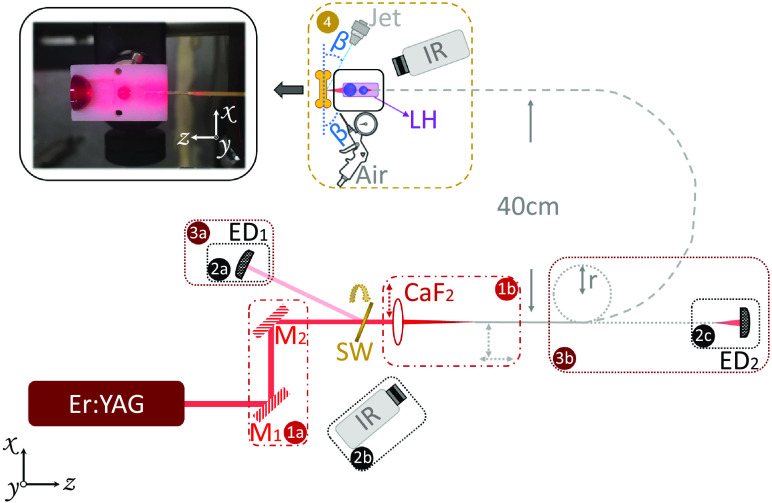
Schematic of the setup used for studying the performance of several glass fibers for transmitting Er:YAG laser light. The laser was coupled to an optical fiber by means of two coupling mirrors (1a) and a coupling ${\mathrm{CaF}}_{2}$ lens (1b). A portion of the initial energy was measured with an energy meter ${\mathrm{ED}}_{1}$ (2a). The transmitted energy was measured with an energy meter ${\mathrm{ED}}_{2}$ (2c). While the reflected and the transmitted energy were being measured, an IR camera recorded the temperature of the fiber input face (2b). The bending stability of the fibers was tested by bending the fibers to different radii r (3b), the transmission efficiency was also determined by the reflected energy measured with ${\mathrm{ED}}_{1}$ (3a), and the transmitted energy measured with ${\mathrm{ED}}_{2}$ (3b). The ablation performance of the laser through one of the fibers was studied (4). An IR camera was used as a temperature feedback system to automatically control a water jet irrigation system. The water jet (diameter 50  μm) was placed at an angle (β=30  deg) in the upper right quadrant of the XZ-plane. A pressurized air gun was placed at an angle (β=30  deg) in the lower right quadrant of the XZ-plane. The photograph next to (4) presents the final lenses and holder design used for ablation.

Figure 2 shows a diagram of the setup used for our study. The setup diagram is divided into four parts, representing the sequence of steps performed during our experiments. The coupling process corresponds to steps (1a) and (1b). Two coupling mirrors $M_{1}$ and $M_{2}$ (1a) were used to optimize the coupling process. In (1b), a calcium fluoride (${\mathrm{CaF}}_{2}$) focusing lens with 25.4-mm focal length was mounted to an XY translating lens mount. The optical fiber was inserted in a fiber chuck for bare fibers (Newport, FPH-DJ), placed on a Z translating mount and an $XYZ\theta_{x}\theta_{y}$ fiber optic positioner (Newport, FP-2A). With a laser $M^{2}$ of 22, the lens provided a beam size of 292  μm at the focal plane. The divergence of the beam was θ=0.14  rad, which fulfilled the requirements of the germanium and the fluoride fibers; it was a little high for the sapphire fiber whose numerical aperture (NA) was 0.12, and too high for the hollow-core fiber (NA=0.025). In previous experiment,^21^ a beam size of 328  μm and a suitable beam divergence of 0.12 rad for the sapphire fiber was used; however, the fiber could not withstand more than 300 mJ. According to the following equation for calculating the beam size $D_{0}$,

$$D_{0}=\frac{2\lambda M^{2}}{\pi \theta },$$ (1)

to satisfy θ≤NA for the hollow-core fiber with the current laser, the beam size at the tip of the fiber had to be ≥1.6  mm, exceeding the size of its 500-μm hollow core. These facts regarding the sapphire and the hollow-core fibers were taken into account in the subsequent experiments.

### 2.3 Transmission Efficiency over Energy

In this section, we describe the transmission efficiency over energy for each fiber for 100 pulses at 5 and 10 Hz. To determine the ratio of transmitted energy over input energy, we used two energy meters ${\mathrm{ED}}_{1}$ and ${\mathrm{ED}}_{2}$, depicted in Fig. 2. To determine the input energy, we used a sapphire window (SW) to reflect a small percentage of the beam into the first energy detector ${\mathrm{ED}}_{1}$, step (2a). The reflected percentage from the SW was about 0.5%. The transmitted energy was measured directly with the second energy detector ${\mathrm{ED}}_{2}$, step (2c).

### 2.4 Transmission Stability over Time and Temperature Monitoring

At the maximum level of energy transmitted by each fiber at 5 and 10 Hz, we studied the stability of the transmission over 5 min. While measuring the reflected and transmitted energies [Fig. 2, steps (2a) and (2c), respectively], we monitored the temperature at the fiber input tip, using an IR camera, FLIR A655sc at 50 fps, step (2b).

### 2.5 Bending Stability

Fiber flexibility and transmission efficiency were studied by bending the fiber to different radii r, as depicted in Fig. 2, until reaching the minimum bending radius reported by the manufacturer for each fiber (Table 1). The two detectors, ${\mathrm{ED}}_{1}$ in step (3a) and ${\mathrm{ED}}_{2}$ in step (3b), were used to determine the transmission efficiency of the bent fiber over time. The measurement was performed for 5 min at each bending radius.

### 2.6 Miniaturized Focusing System for Appropriate Ablation through Fiber

The ablation of bone in free space, using our Er:YAG laser, had already been optimized for a beam size of 526  μm.^11^ We designed a lens system capable of focusing the beam in a manner similar to that of the previous study’s free-space setup.^11^ Since beam propagation differs depending on the type of fiber it passes through (due to different output NA), the lens was designed for the fiber that we considered most suitable for endoscopic laser surgeries, based on the experimental findings. Figure 2, step (4) shows a picture of the lens design used for the ablation experiments. The design is described in more detail in Sec. 3.4.

### 2.7 Bone Ablation Performance in Minimally Invasive Settings

Figure 2, step (4) depicts the bone ablation process. The samples used were sheep tibia cortical bone from the Musculoskeletal Research Unit at the University of Zürich. The cadaveric sheep bones were obtained from an animal euthanized in the context of an experiment unrelated to the study reported herein. The cadaveric samples were used within 84 h after the animal was euthanized, and kept moist at all times before ablation. Three ablation lines, 10-mm long, were each created over a 4-min period. The irrigation system was composed of a Maximator pump ref No. MP030066 and a nozzle from Synova Laser MicroJet Technology. The nozzle produced a water jet with a diameter of 50  μm. For the experiments, the pump ran at 30 bar pressure. Pressurized air of 2 bar was used to blow off the debris and the remaining water from the ablated area. The IR camera described in Sec. 2.4 was used to monitor the superficial temperature of the bone. Immediately after ablation, the samples were preserved in 4% formalin. One week later, the samples were taken out for a short time to measure the depth and width of the ablated areas. This measurement was performed using an optical coherence tomography (OCT) system available in our laboratory. The OCT system was composed of an Axsun swept laser with a 1064-nm central wavelength and a 100-nm bandwidth. B-scans to analyze the images were taken with a 14.5-mm field of view and 3.51-mm imaging depth.

The ablation setup used in our study, comprising an automated temperature feedback mechanism (IR camera) for the irrigation system (thin water jet) and pressurized air, was described in detail in our previous study.^11^

### 2.8 Histology

Sheep cortical bone samples were used to create cuts using the Er:YAG laser through an optical fiber. The non-decalcified bone samples were fixed in 4% buffered formalin, then dehydrated in an ascending series of ethanol, after which, the samples were defatted in xylene and immersed in methylmethacrylate under vacuum; final polymerization was reached after complete infiltration. Thick sections (200 to 700  μm) were cut using a diamond saw (Exact^®^ 310 saw), mounted on Acropal slides and surface stained with toluidine blue. The sections were then imaged using a macroscope (Z6 APO A, Leica Microsystems) with a DFC450 Digital Camera (Leica Microsystems). For higher magnifications, we used a DMR microscope system (Leica Microsystems) with a DC320 digital camera (Leica Microsystems).

## 3 Results and Discussion

### 3.1 Transmission Efficiency over Energy

**Fig. 3:**
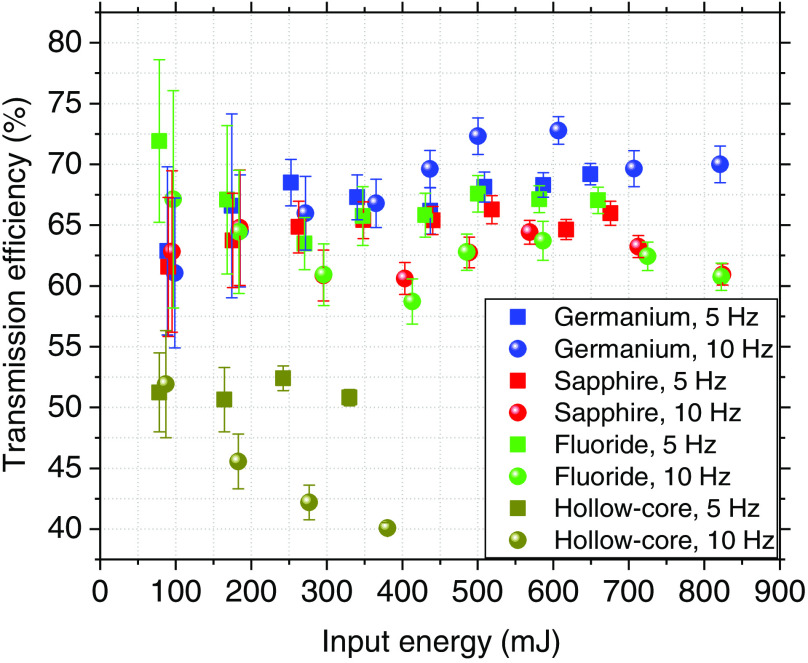
Transmission efficiency of germanium oxide, sapphire, zirconium fluoride, and hollow-core silica fibers at 5 and 10 Hz at different input energies.

Figure 3 shows the transmission efficiency over the input energy of the laser, obtained for each fiber at 5 and 10 Hz. Each data point on the graph is the average over 100 pulses with the corresponding standard deviation.

During our experiments, the transmission efficiency obtained for the sapphire fiber was 64%±2%; however, other researchers have shown that the sapphire fiber can reach up to 90% transmission efficiency.^18^ The relatively low performance in our study was most likely caused by a beam divergence that did not completely fulfill the NA requirements of the sapphire fiber. As seen in Fig. 3, the fluoride fiber achieves higher transmission at low energies and then tapers off; its transmission efficiency was 65%±3%. The hollow-core fiber tip burned at energies higher than 400 mJ, resulting in the need to unmount and polish the fiber. The maximum input energy for the hollow-core fiber (without burning) was 330 and 380 mJ at 5 and 10 Hz, respectively. The lowest transmission was observed in the hollow-core fiber; at 5 Hz, the transmission efficiency was only 51%±1%, while at 10 Hz, the transmission was 45%±5%, decreasing over the energy. NA matching issues account in part for the low performance of the hollow-core fiber. Of the fibers used, the germanium fiber showed the highest transmission efficiency at 68%±3%, similar to that achieved in other studies.^22^

### 3.2 Transmission Stability over Time and Monitoring the Input Tip Temperature

After examining the behavior of the fibers exposed to different input energies, the transmission stability of the fibers was tested for 300 s (5 min). We applied the laser’s maximum achievable energy level, about 820 mJ, to the input fiber face. Since the hollow-core fiber could not withstand such high energy levels, we used energy settings of 330 and 380 mJ at 5 and 10 Hz, respectively, for this fiber.

**Fig. 4:**
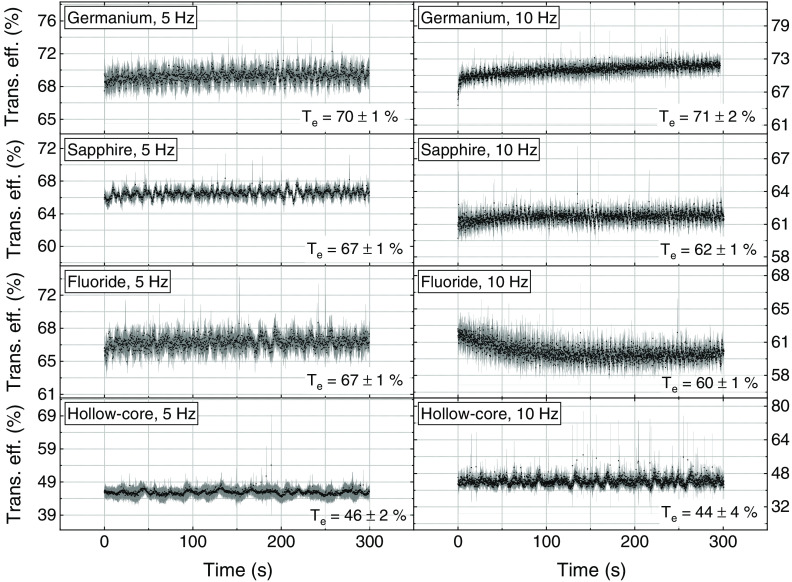
Transmission efficiency over time of each fiber at 5 and 10 Hz. The input energy was 820 mJ for all fibers except the hollow-core fiber, which was set at 330 and 380 mJ at 5 and 10 Hz, respectively.

Figure 4 shows the evolution of the transmission efficiency of each fiber over 5 min at 5 and 10 Hz. The values (black data points) presented in each graph are an average of five individual measurements, the gray areas represent the corresponding standard deviation.

In general, all of the fibers showed stable energy transmission over time. The fluoride fiber showed a decrease in energy transmission in the first 100 s, but stabilized thereafter.

**Fig. 5:**
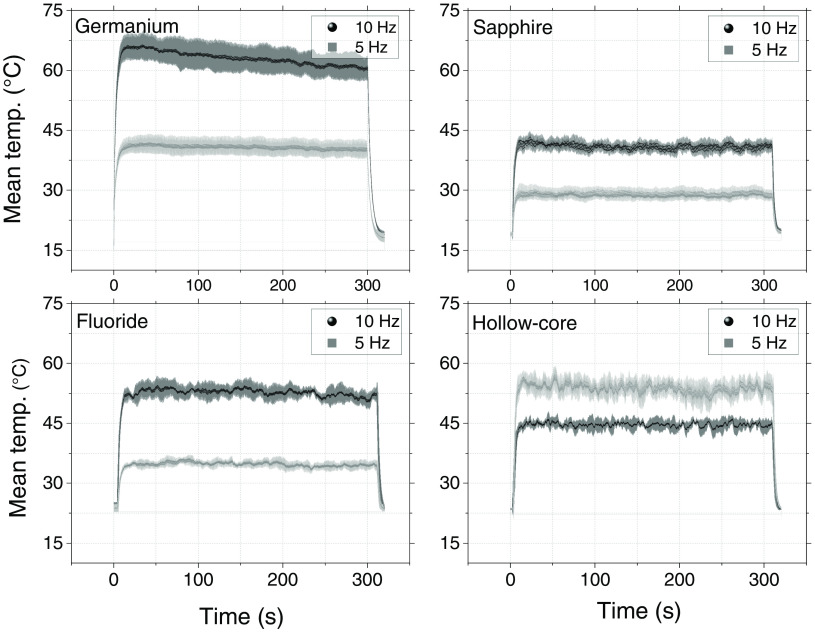
Evolution of the temperature of each input fiber tip, monitored for 300 s. The input energy of the laser was 820 mJ for all fibers except the hollow-core fiber, which was set at 330 and 380 mJ at repetition rates 5 and 10 Hz, respectively.

The input face temperature of each fiber was monitored while simultaneously measuring its energy transmission. Figure 5 shows the average temperature of each fiber tip.

At 10 Hz, the temperature of the germanium fiber dropped within the first 100 s; this is in part because the energy of the laser drops a little over time. The drop in temperature probably also reflects a decrease in the fiber material’s heat capacity around the average temperature of 70°C, measured with our thermal camera. At lower temperatures, there was no noticeable change in the material’s heat capacity, as observed for both the germanium oxide fiber at 5 Hz and for the other fibers. The sapphire fiber tip’s temperature increased the least among all of the fibers. Additionally, sapphire’s physical properties make it highly resistant to scratches and fractures.^23^ Therefore, for endoscopic applications, one may consider using sapphire fibers or splicing them in other fibers.^24^ Doing so may reduce the heat at the endoscope’s tip, thereby also reducing the risk of damaging sensitive material and making it easier to manipulate the fiber.

### 3.3 Bending Stability

After the fibers were tested for transmission efficiency, behavior over time, and input tip temperature over time, we bent the fibers from a straight position up to the minimum bending radii reported by the manufacturers.

**Fig. 6:**
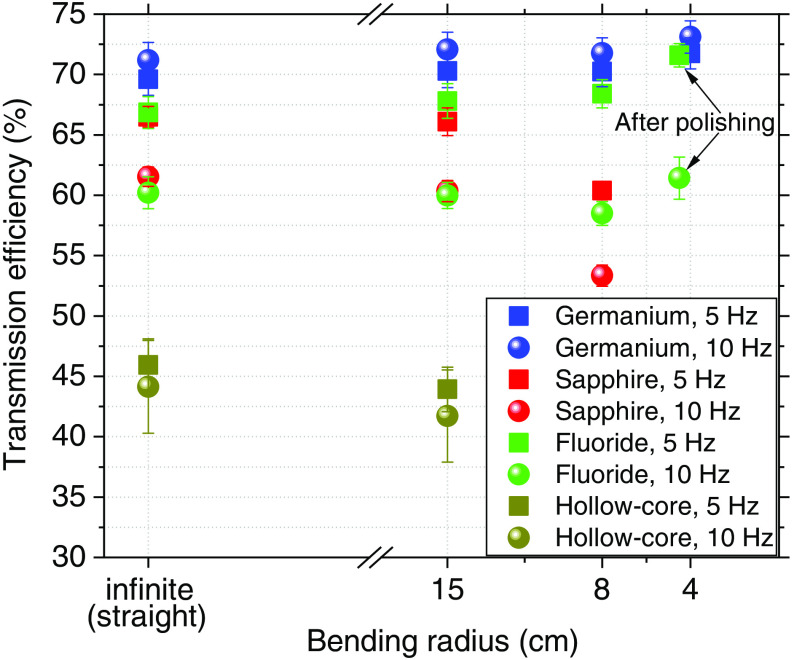
Transmission efficiency of each fiber at different bending radii. The transmission efficiency of the fluoride fiber at 4.5 cm radius corresponds to the response of the fiber after polishing its surface. The input energy of the laser was 330 mJ (at 5 Hz) and 380 mJ (at 10 Hz) for the hollow-core fiber, and 820 mJ for the others. The repetition rate of the laser was 5 and 10 Hz.

Figure 6 shows the effect of bending the fibers on transmission efficiency. As in Sec. 3.2, we delivered the maximum possible energy to the fibers. Each individual data point in the graph is the average over 5 min of five individual measurements.

As seen in Fig. 6, the transmission efficiency of the fibers does not change remarkably when bent to the minimum radius possible, except in the case of the sapphire fiber. At its minimum bending radius, the transmission of the sapphire fiber dropped from 60% to 53% at 10 Hz and from 67% to 60% at 5 Hz. The transmitted energy dropped in an unusual way for the bent fluoride fiber: the fiber tip was burnt and had to be polished. The transmission efficiency of the fluoride fiber at a 4.5-cm bending radius, as reported in Fig. 6, corresponds to the response of the fiber after repair.

### 3.4 Bone Ablation Performance in Minimally Invasive Settings

Based on the properties of the fibers (Table 1) and the studies realized in the previous sections, we found that the germanium oxide fiber fulfilled most of the requirements for use in endoscopic surgical applications. The germanium fiber is biocompatible, showed slightly higher transmission efficiency than the other fibers (Figs. 3 and 4), and is the most flexible among the fibers tested; its minimum bending radius is 4 cm. The only disadvantage of the germanium oxide fiber relative to the others is the high tip temperature reached while the laser was being coupled. However, this issue can be solved by choosing coupling and refocusing materials (for lenses and holders) that dissipate the heat efficiently, or by adding a cooling system to the fiber. The fiber tips can be treated, for instance. Previous studies have shown that the germanium fiber can be spliced with sapphire, although doing so reduced its transmission from 68% to 65%.^22^

**Fig. 7:**
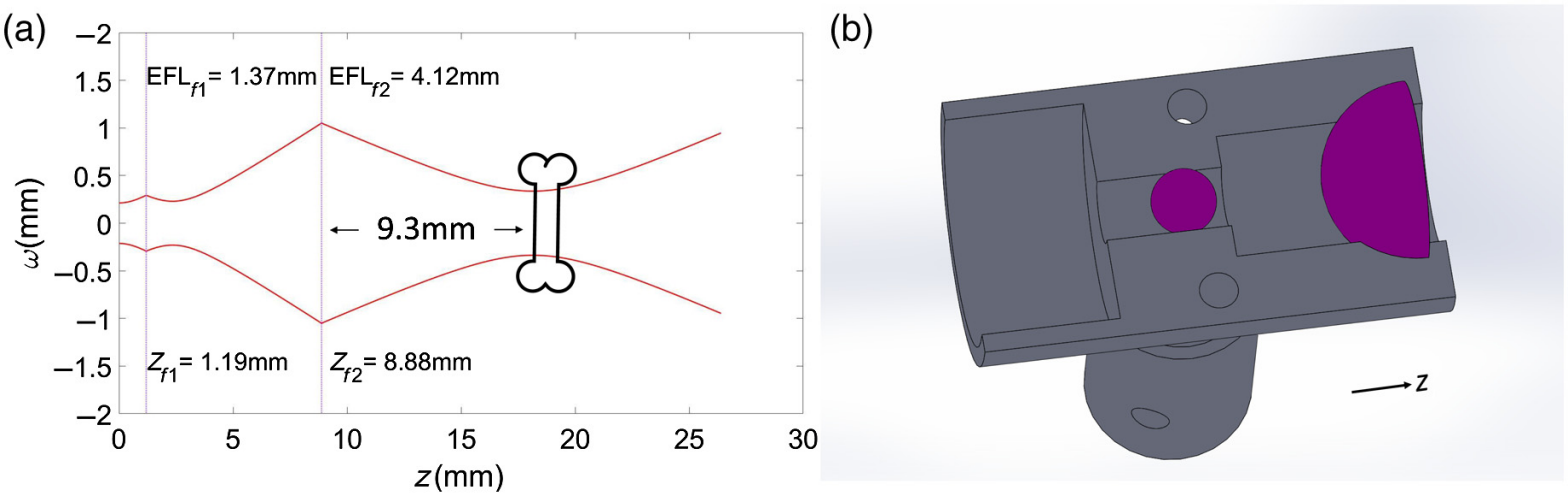
(a) Schematic of the propagation of the beam after the fiber and the two focusing lenses (represented by the two vertical dashed lines) have been inserted, calculated using MATLAB. (b) Lenses and holder design made in SolidWorks.

In this section, we present a refocusing system designed for the germanium fiber. The refocusing system consisted of two lenses inserted into a holder. This system was used for ablating sheep bones. Figure 7 shows a schematic of beam propagation after the fiber and the two focusing lenses have been inserted, such that the beam focuses on the bone surface at almost 10 mm beyond the last lens. The lenses were ruby-doped sapphire ball and half-ball lenses from Edmund Optics. The corresponding effective focal lengths were ${\mathrm{EFL}}_{f1}=1.37\;\mathrm{mm}$ and ${\mathrm{EFL}}_{f2}=4.12\;\mathrm{mm}$. The first lens was a ball lens with a diameter of 2.38 mm, while the second lens was a half-ball lens with a diameter of 6.35 mm. This lens system provides a beam diameter of 673  μm at the focal plane, located 9.3 mm away from the last lens.

The lens holder was 3D-printed in VeroWhitePlus, RGD835 material. This material is not biocompatible and, therefore, not recommended for real surgery applications. However, it was a very useful prototype and served to identify the benefits of our system, as well as the issues to be improved.

**Fig. 8:**
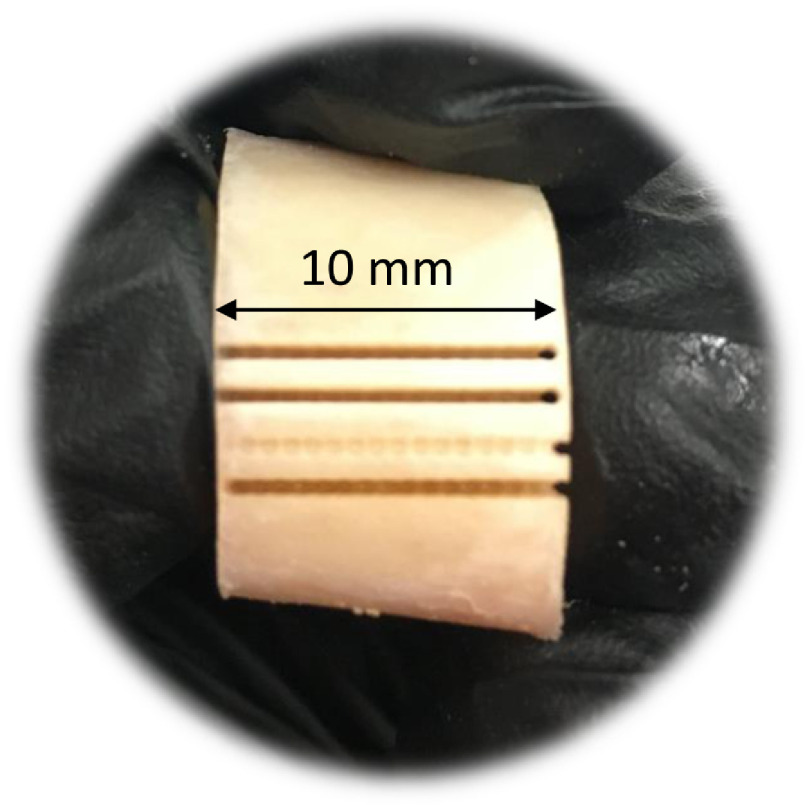
Photograph of one of the sheep bones ablated with the Er:YAG laser through a germanium oxide fiber. All of the lines have a length of 10 mm. The first two lines and the fourth one were made by scanning the bone 180 times (about 4 min at a speed of 8  mm/s). The third line was ablated with only one laser scan.

Figure 8 shows a photograph of a sheep bone ablated with the Er:YAG laser coupled with the germanium oxide fiber. For ablation, the maximum achievable energy of the laser beyond the lenses was 370 mJ at a 10-Hz repetition rate. The ablation depth achieved through the germanium oxide fiber was 6.82±0.99  mm, while the width was 0.69±0.06  mm.

### 3.5 Histology Analysis

**Fig. 9:**
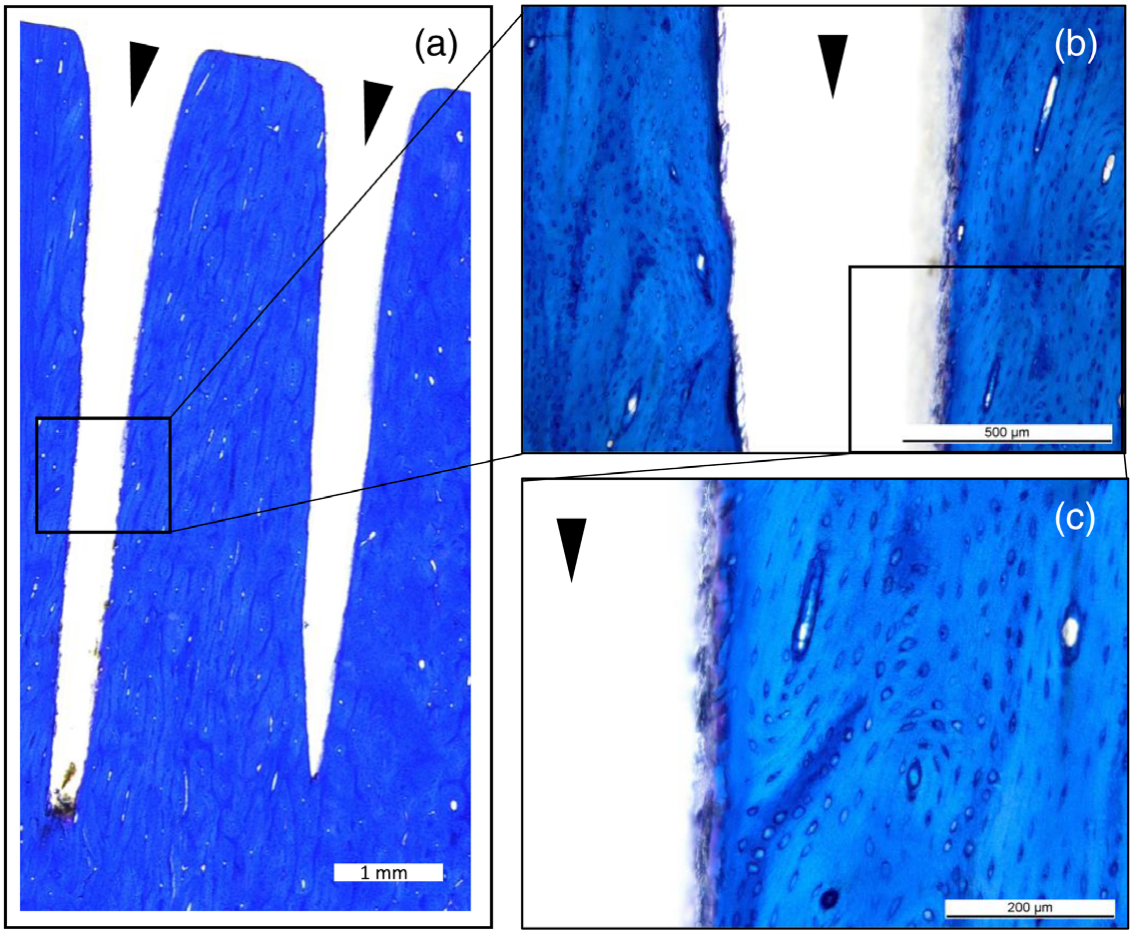
Sheep cortical bone with two cuts made with the Er:YAG laser through ${\mathrm{GeO}}_{2}$ fiber, shown in panel (a). Higher magnifications in panel (b) and panel (c) show the bone tissue interface with the laser’s path, as well as the tissue structure. The toluidine blue stain highlights the cells within the bone and the lamellar structure, and shows how the laser can cut through these structures in a controlled manner. The black arrows indicate the laser cuts. The scale bars indicate 1 mm, 500  μm, and 200  μm in panels (a), (b), and (c), respectively.

As shown in Fig. 9, the laser was able to cut through sheep cortical bone in a straight path through the bone lamellae. The structural features of the cortical bone adjacent to the laser path remained intact, as seen in Fig. 9(c).

## 4 Conclusion

Four fibers suitable for transmitting high-power laser energy in the mid-IR region of the spectrum were used for coupling an Er:YAG laser at a wavelength of 2.94  μm. The setup was not optimal for the sapphire and hollow-core fibers, since the divergence of the beam did not fully match the required NA. However, the sapphire fiber remained undamaged at the laser’s maximum energy of 820 mJ. The hollow-core fiber could only withstand a maximum energy of 380 mJ, and the germanium fiber had the highest transmission efficiency at 68%±3%, as shown in Fig. 3. Transmission efficiency was stable for 5 min at 820 mJ for all but the hollow-core fiber, which was stable at 380 mJ. However, the fluoride fiber showed a drop in efficiency after the first 100 s (Fig. 4). Figure 5 shows the input tip temperature over time. In general, it was stable for all fibers, however, the maximum average temperature of the germanium fiber’s tip showed higher temperature around 70°C compared to the other fibers. When decreasing the bending radius of the fluoride fiber to 4.5 cm, we observed a drop in the transmitted energy (Fig. 6) and an increase in the temperature of the tip. Examination of the fiber surface through a fiber microscope confirmed the suspicion that the fiber had been damaged. Based on the reported results, and on the results from our previous studies,^21^ we consider the germanium oxide fiber to be the most appropriate choice for future endoscopic applications. Still, the temperature issue must be resolved, for instance, by splicing the end tips with sapphire. We designed a lens system capable of focusing the laser beam after the fiber, at nearly 10 mm beyond the last lens, with a beam diameter of 673  μm. At the maximum achievable output energy of 370 mJ at 10 Hz, we obtained an ablation depth of 6.82±0.99  mm in sheep bone. At present, the small working space of the fiber setup limits our ability to align the water jet and pressurized air with the laser beam. Therefore, the irrigation and cleaning design must be adapted to small working distances of less than 10 mm. Histology image analysis showed no structural damage on the surroundings of the cut made with the Er:YAG laser through the ${\mathrm{GeO}}_{2}$ fiber (Fig. 9). The fiber system presented in this study has the potential to be implemented in endoscopic laserosteotomy systems.

## Code, Data, and Materials Availability

The data generated and analyzed in this study are included in this paper.
