# Supplementary figures and images for: Optical fibers for endoscopic high-power Er:YAG laserosteotomy
Source: J Biomed Opt. 2021 Sep 13;26(9):095002. doi: 10.1117/1.JBO.26.9.095002 (PMC8435982; doi:10.1117/1.JBO.26.9.095002)

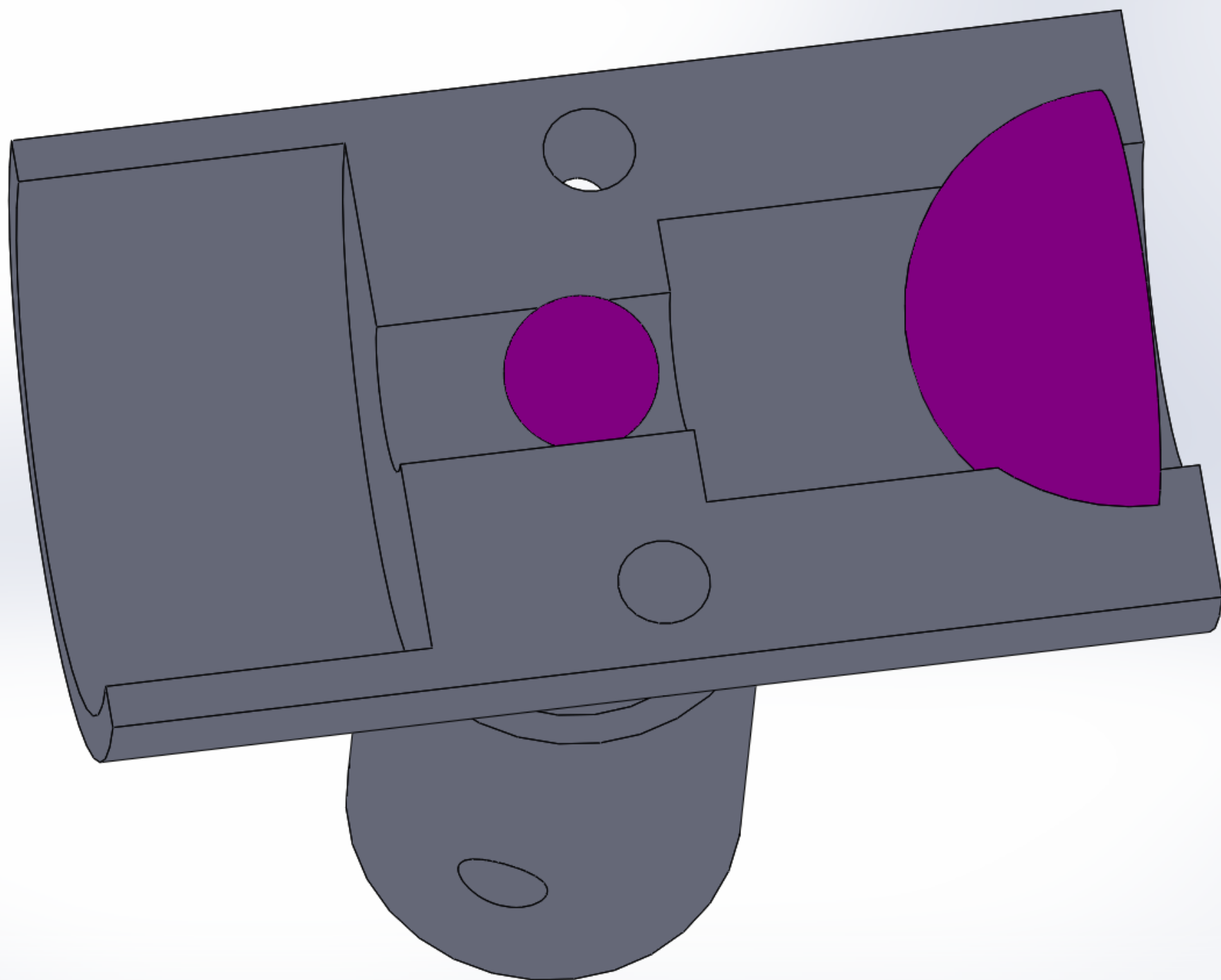

Supplement: Supplementary file 1 [file JBO_026_095002_SD001.pdf]
